# Water contamination and cytogenetic effects in fish from coastal lagoons of southern Brazil

**DOI:** 10.1007/s10661-026-15335-9

**Published:** 2026-04-27

**Authors:** Eloisa Bianchi, Bárbara Tamires da Silveira, Marina Becker Klein, Jenifer Panizzon, Cacinele Mariana da Rocha, Larissa Schemes Heinzelmann, Luciano Basso da Silva

**Affiliations:** 1 Feevale University, ERS-239, 2755, Novo Hamburgo, Rio Grande do Sul, 93525-075 Brazil; 2 Centro de Estudos Costeiros, Limnológicos e Marinhos (CECLIMAR), Universidade Federal do Rio Grande do Sul (UFRGS), Avenida Tramandaí 976, Imbé City, Rio Grande do Sul Brazil

**Keywords:** Genotoxicity, Water quality, Environmental monitoring, Sentinel species

## Abstract

Coastal lagoons are ecologically sensitive environments increasingly exposed to human pressures, particularly in regions affected by seasonal tourism. These pressures may alter water quality and induce sublethal biological responses in aquatic organisms. This study evaluated physicochemical and microbiological parameters and cytogenetic biomarkers in the fish *Geophagus iporangensis* from nine lagoons within the Tramandaí River Basin (TRB), southern Brazil, sampled before, during, and after the summer vacation period. Water samples were analyzed for dissolved oxygen (DO) and thermotolerant coliforms (TC), while micronuclei (MN) and nuclear abnormalities (NA) were assessed in fish erythrocytes. DO concentrations in all samples complied with standards for the highest water quality class. In contrast, TC levels exceeded limits for primary contact recreation in eight lagoons in at least one sampling event, and four lagoons remained above these limits throughout all sampling periods. MN frequencies showed no significant spatial or temporal variation. NA frequencies ranged from 1.3 to 12.3‰ and exhibited significant spatial and temporal variability among lagoons. Seasonal population increases during the vacation period coincided with elevated fecal contamination in two lagoons and higher NA frequencies in four lagoons; however, no consistent seasonal pattern was observed across the study area. Moreover, no clear association was detected between TC concentrations and cytogenetic damage. These findings indicate that genotoxic responses in the TRB lagoons are influenced by lagoon specific and potentially diffuse contamination sources rather than by seasonal population increases alone. The co occurrence of fecal contamination and genotoxic responses raises concerns regarding environmental quality in coastal tourist areas.

## Introduction

Coastal zones encompass a wide variety of natural ecosystems and resources, making them highly attractive for human activities such as settlement and recreational tourism (Stanchev et al., 2015). Coastal lagoons are increasingly affected by untreated sewage and the discharge of domestic, agricultural, and industrial waste, leading to the degradation of water quality in these ecosystems (Celma et al., 2022; Redwan & Elhaddad, 2022). The coastal plain of Rio Grande do Sul state extends approximately 640 km along the southern coast of Brazil, with an estimated total surface area exceeding 37,000 km^2^, of which 14,260 km^2^ corresponds to lagoons, ponds, and rivers. This ecosystem includes numerous shallow lagoons that run parallel to the ocean coast and are interconnected by canals and small rivers, forming the extensive Tramandaí River Basin (TRB) (Schwarzbold & Schäfer, 1984). This and other coastal basins in southern Brazil support a highly endemic fish fauna, with some lagoons serving as reproduction, feeding, and nursery grounds for several fish species (Langeani et al., 2009). In recent decades, regional population growth and economic activities within the TRB, particularly irrigated agriculture, have intensified, leading to increased water consumption and exerting greater pressure on natural water resources (Loitzenbauer & Mendes, 2012).

The presence of xenobiotics in aquatic environments and their genotoxic effects on biota are of significant relevance to environmental health (Andrés et al., 2025). DNA-damaging agents are implicated in various pathological processes, including carcinogenesis and reproductive disorders (Kovacik & Helczman, 2025). Fish species are widely used as sentinel organisms in aquatic toxicity studies (Ahmed et al., 2020), and the micronucleus (MN) test in fish erythrocytes has been successfully applied to assess the genotoxic impact of environmental pollutants under field conditions (Bolognesi & Hayashi, 2011; Marques et al., 2025; Udroiu, 2006). Micronuclei form during cell division due to aneugenic events (chromosome loss) associated with spindle apparatus dysfunction or clastogenic events (chromosome breakage) (Udroiu, 2006). The concurrent analysis of erythrocyte nuclear abnormalities (NA), such as nuclear buds, nuclear invaginations, and binucleated cells, provides an additional biomarker for toxicity assessment (Pacheco & Santos, 1998; Çavas and Könen, 2008; Omar et al., 2012).

The *Geophagus brasiliensis* species complex (Cichlidae) is widely distributed, ranging from the coastal basins of Bahia State in northeastern Brazil to coastal rivers in eastern Uruguay, as well as parts of the São Francisco and La Plata rivers basins (Kullander, 2003). In addition to its ecological relevance, this species complex is also used as a food source by local populations (de Jesus et al., 2016). According to Argolo et al. (2020), *G. iporangensis* (Haseman, 1911) is the most widely distributed species within the complex, occurring throughout southern coastal basins. *G. iporangensis* is an omnivorous bottom-dweller with diurnal habits, a preference for lentic environments, and territorial behavior (Mazzoni & Iglesias-Rios, 2002). Specimens from this species complex have been successfully employed in laboratory toxicity assessments of environmental pollutants, including wastes from ornamental stone processing (Venturoti et al., 2019), cadmium exposure (Queiroz et al., 2019), and cyanotoxins (Calado et al., 2020). In addition, several *in situ* studies conducted in southern Brazil and neighboring regions have evaluated MN and NA frequencies in fish exposed to a range of anthropogenic pressures, further demonstrating the robustness of these cytogenetic biomarkers for environmental monitoring. For example, Beninca et al. (2012) documented chronic genotoxic damage in *G. brasiliensis* inhabiting estuarine lakes in Santa Catarina State, whereas Gomes et al. (2019) observed significant DNA damage in this species following exposure to metal-contaminated waters in Minas Gerais. Within the Upper Paraná River basin, Viana et al. (2017) reported pronounced mutagenic and genotoxic effects in native fish from the Amambai River, accompanied by substantial accumulation of non-essential metals in liver tissues. Similarly, in neighboring Uruguay, assessments of coastal and estuarine fish have revealed elevated MN and NA frequencies in areas influenced by urban and industrial discharges (Gutiérrez et al., 2015). Collectively, these studies highlight the regional relevance of cytogenetic biomarkers and underscore the need for expanded assessments in the coastal lagoons of southern Brazil, where such data remain notably scarce.

The population of coastal towns within the TRB region totals 261,346 inhabitants, with significant seasonal influxes during peak vacation periods. The summer season, which lasts from December to March, can see population increases of over 400% in some municipalities (Comitê de Gerenciamento da Bacia Hidrográfica do Rio Tramandaí, 2013). Seasonal population surges can overload sewage treatment systems, causing significant deterioration in water quality (Ben-Haddad et al., 2023). Domestic sewage is a major source of pollution, introducing emerging contaminants such as pharmaceuticals, pesticides, and heavy metals, while inadequate treatment increases health risks and the spread of waterborne diseases (Novaes Matilde et al., 2025; Tagar et al., 2025). Therefore, beyond microbiological hazards, sewage effluents contain pollutants that can induce genotoxic effects in aquatic organisms (Freitas et al., 2023; Mishra et al., 2023). In this context, thermotolerant coliforms serve as key indicators for assessing sewage contamination in surface waters (Tagar et al., 2025).

Although sewage discharges represent a major source of contamination in the TRB, they constitute only one component of a broader and more complex pollution scenario. Previous studies in the region have documented the presence of multiple genotoxic contaminants, including metals such as cadmium, chromium, mercury, and lead in fish tissues (Tesser et al., 2021), as well as a range of pesticides associated with agricultural runoff (Pagani et al., 2022). More recently, the detection of microplastics in TRB environments (Fonseca et al., 2025) has further highlighted the diversity of pollutant sources affecting these ecosystems. The coexistence of these contaminants,originating from urban effluents, agricultural activities, industrial residues, and emerging pollutants,reinforces the need for a comprehensive interpretative framework. Such complexity underscores the importance of using sensitive genotoxic biomarkers to detect sublethal biological effects that may not be apparent through traditional water quality analysis.

Although the general mechanisms and applications of genotoxic biomarkers in fish are well established, their effective use in environmental assessments requires contextualization within specific ecological settings. Despite the ecological and socioeconomic significance of the TRB, information on chronic or seasonal genotoxic pressures acting on its aquatic biota remains scarce. To date, only one study has evaluated genotoxic effects in fish species within the coastal plain of Rio Grande do Sul. De Andrade et al. (2004) investigated two native estuarine species—the gray mullet (*Mugil* sp.) and the sea catfish (*Netuma* sp.)—in the Tramandaí and Mampituba Rivers, and reported increased DNA damage potentially linked to seasonal population surges in nearby towns during warmer months. However, the lagoons of the TRB have not yet been assessed for the presence of genotoxic substances, and it remains unknown whether seasonal increases in human occupation, wastewater discharge, and diffuse pollution within this basin translate into measurable cytogenetic alterations in resident fish populations.

In light of this knowledge gap, the present study aimed to evaluate cytogenetic damage in *G. iporangensis* from nine coastal lagoons and to investigate whether these effects are associated with peak vacation periods. Based on the marked seasonal variability in population density and wastewater generation in the region, we hypothesized that (i) thermotolerant coliform concentrations would increase during the summer vacation period due to intensified domestic sewage loads and (ii) fish collected during this period would display higher frequencies of MN and NA relative to pre-vacation and post-vacation months. We further hypothesized that lagoons with persistently elevated fecal contamination would exhibit correspondingly higher cytogenetic damage, reflecting chronic exposure to genotoxic pollutants.

## Material and methods

**Fig. 1:**
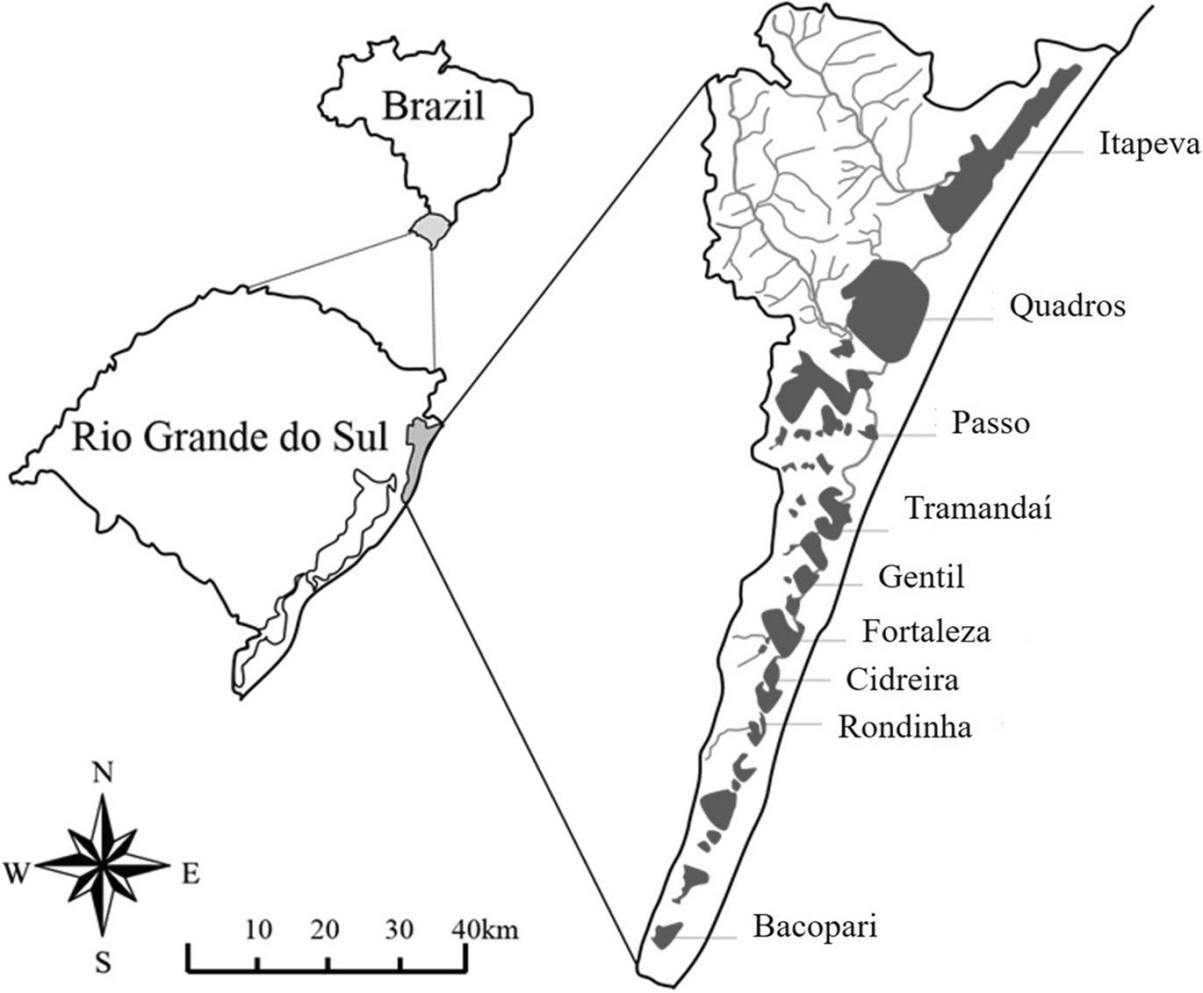
Study area. Localization of the nine coastal lagoons studied in the Tramandaí River Basin (Rio Grande do Sul state, Brazil)

The Tramandaí River Basin (TRB) is located in the northeastern part of Rio Grande do Sul State, southern Brazil, between the geographic coordinates 29°17′ to 30°18′ S latitude and 49°44′ to 50°24′ W longitude. The TRB encompasses 21 municipalities and has a drainage area of 2,978.11 km^2^ and a 150 km-long coastal strip, extending from Itapeva Lagoon in the north to Bacopari Lagoon in the south (Comitê de Gerenciamento da Bacia Hidrográfica do Rio Tramandaí, 2013). The basin contains 41 lagoons, many of which are interconnected by rivers and channels that ultimately discharge into a single outlet, the Atlantic Ocean, through the Tramandaí Lagoon (Schwarzbold & Schäfer, 1984). Nine lagoons were selected within the TRB along approximately 150 km of the coastal plain, being in the north–south direction (Fig. 1): Itapeva (29°32′14, 8″S/49°56′12″O), Quadros (29°45′54, 1″S/50°04′40,5″O), Passo (29°56′51, 9″S/50°06′3, 5″O), Tramandaí (29°56′51, 9″S/50°08′57,2″O), Gentil (30°03′3, 5″S/50°11′31, 3″O), Fortaleza (30°08′47, 2″S/50°13′8, 2″O), Cidreira (30°10′7, 6″S/50°14′45″O), Rondinha (30°13′30, 3″S/50°15′42, 7″O), and Bacopari (30°31′42, 2″S/50°25′31″O). According to the Köppen classification system, the area is characterized by a humid subtropical climate, with an average annual temperature of around 20 °C and rainfall of about 1300 mm (Salomoni, 1997).

Adult specimens of *G. iporangensis* (Length: 7.3 ± 1.2 cm) were collected using gillnets during three distinct periods: November 2012 (pre-vacation), January 2013 (peak vacation), and March 2013 (post-vacation). A total of 27 sampling events were conducted (three campaigns in each of the nine lagoons) and *G. iporangensis* specimens were successfully captured in 25 of these samplings (samples from Rondinha in November and from Cidreira in March were not obtained and, therefore, were excluded from the statistical analysis). The sex of the fish was not determined during sampling.

Peripheral blood samples of fish were obtained from the gills using a surgical syringe. Blood smears were prepared on clean microscope slides, dried at room temperature, and then fixed in absolute ethanol for 10 min. Slides were stained with 5% Giemsa for 10 min. Slides were coded and MNs were analyzed according to the criteria described by Al-Sabti and Metcalfe (1995). A total of 2000 erythrocytes per fish were analyzed using an Olympus BX41 light microscope at 1000 × magnification. Additional nuclear abnormalities, such as invaginations, buds, and binucleated cells, were assessed following the methodology described by Carrasco et al. (1990). These abnormalities were collectively classified as nuclear abnormalities (NA) and scored together (Seriani et al., 2015; Vieira et al., 2016). This study was approved by the Ethics Committee for Animal Experimentation of Universidade Feevale (Protocol No. 02.13.022).

In addition, one surface water sample was collected at each sampling site, always at the same location where gillnets were installed for fish sampling. No visible sewage discharge was observed at these points. Water samples were collected and transported to the laboratory in accordance with the Standard Methods for the Examination of Water and Wastewater (APHA, 2012). Analyses were performed at the Coastal and Limnological Studies Center of the Federal University of Rio Grande do Sul (CECLIMAR – UFRGS). Dissolved oxygen (DO) was determined using the Winkler method, and thermotolerant coliforms were analyzed using the Membrane Filtration (MF) technique. Results for thermotolerant coliforms were expressed as Most Probable Number (MPN) per 100 mL (MPN/100 mL). Other physicochemical parameters were not analyzed due to logistical constraints related to the volume of water required for transport or because the laboratory was not equipped to perform these tests. The measured values of DO and thermotolerant coliforms were compared against the limits established by Brazilian environmental legislation.

Normality and homogeneity of variance for biomarker frequencies were assessed using the Shapiro–Wilk and Levene tests, respectively. The data were not transformed prior to analysis. Because the data did not meet the assumptions of normality, comparisons among lagoons, as well as among sampling periods within each lagoon, were performed using the Kruskal–Wallis test (with Bonferroni correction), followed by Dunn’s multiple comparison test when appropriate. When significant differences were detected using the Kruskal–Wallis test, the effect size was calculated as ordinal eta-squared [η^2^ₕ = (H – k + 1)/(n – k); where H is the Kruskal–Wallis test statistic, k is the number of groups, and n is the total number of observations] (Fiel Peres, 2026). Spearman correlation analysis was conducted to evaluate the relationship between thermotolerant coliform concentrations and the frequencies of the genotoxicity biomarkers. All analyses were performed using the Statistical Package for the Social Sciences – SPSS 22 considering a significance level of *p* ≤ 0.05.

## Results

The results of dissolved oxygen (DO) and thermotolerant coliforms (TC) from the 27 water samples collected across the nine lagoons of the Tramandaí River Basin (TRB) are presented in Table 1. Dissolved oxygen (DO) concentrations in all samples met the Brazilian environmental standards for Class I freshwaters, which require a minimum of 6 mg/L, as established by CONAMA Resolution No. 357/2005. Class I waters are considered high-quality and suitable for primary contact recreation (e.g., swimming, water skiing, diving), irrigation of vegetables and fruits consumed raw, aquaculture, fishing, and human consumption after simplified treatment (Brasil, 2005). Thermotolerant coliform concentrations exceeded the permissible limit established by CONAMA Resolution No. 357/2005 (1000 MPN/100 mL for Class I waters, suitable for primary contact recreation) in 19 of the 27 water samples analyzed. Elevated TC levels were detected in all samples from the Quadros, Tramandaí, Gentil, and Cidreira lagoons. Furthermore, two samples from each of the Passo, Fortaleza, and Rondinha lagoons, as well as one sample from the Bacopari lagoon, also surpassed the legal threshold. In contrast, all three samples from the Itapeva lagoon complied with the recommended limits.

**Table 1:** Values of dissolved oxygen (DO mg L^−1^) and thermotolerant coliforms (MPN/100 mL) in water samples of nine lagoons of the TRB

Parameter (limits)	Sample	Itapeva	Quadros	Passo	Tramandaí	Gentil	Fortaleza	Cidreira	Rondinha	Bacopari
DO	November	8.4	6.4	8.1	10.2	8.2	7.9	7.6	6.9	9.5
(> 6 mg L^−1^)^a^	January	8.2	8.1	9.4	7.3	6.9	7.8	8.4	6.8	9.5
	March	7.5	8.0	7.5	7.3	7.7	8.0	7.7	7.9	10.6
Thermotolerant coliforms	November	410.6	**2419.6**	365.4	> 2419.6	**> 2419.6**	648.8	**> 2419.6**	**1203.3**	20.2
(< 1000 MPN/100 mL)^b^	January	866.4	**> 2419.6**	**> 2419.6**	**> 2419.6**	**> 2419.6**	**1553.1**	**1986.3**	**1413.6**	125.9
	March	248.9	**1732.9**	**> 2419.6**	**> 2419.6**	**> 2419.6**	**1203.3**	**1986.3**	435.2	**1203.3**

^a^CONAMA 357/2005, values for class I water quality. ^b^CONAMA 357/2005, satisfactory water quality for recreational use (class I water quality). Values above the limits are highlighted in bold

**Fig. 2:**
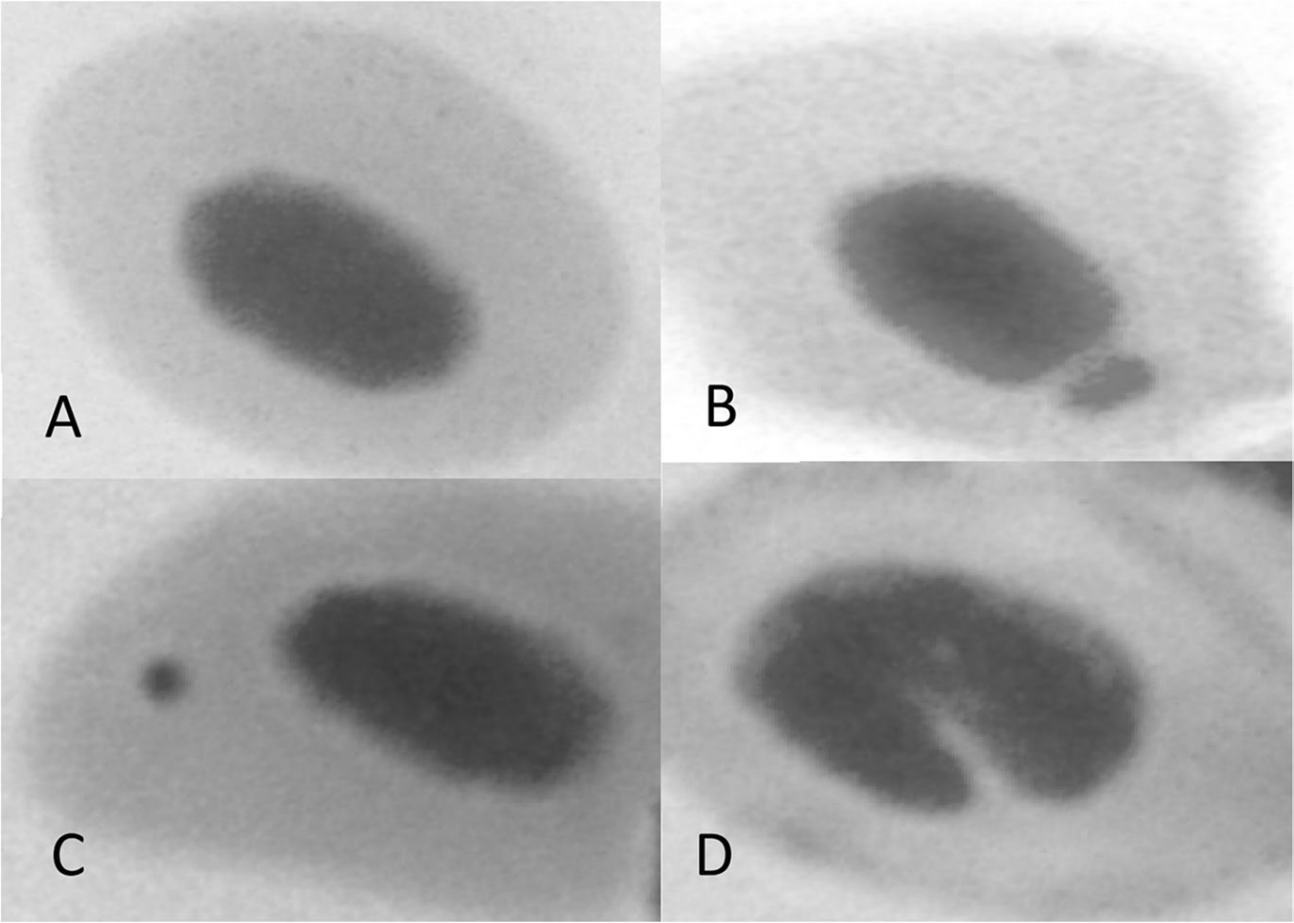
Images of erythrocytes from *G. iporangensis*: (**A**) normal cell; (**B**) nuclear bud; (**C**) micronucleus; (**D**) nucleus with invagination

**Fig. 3:**
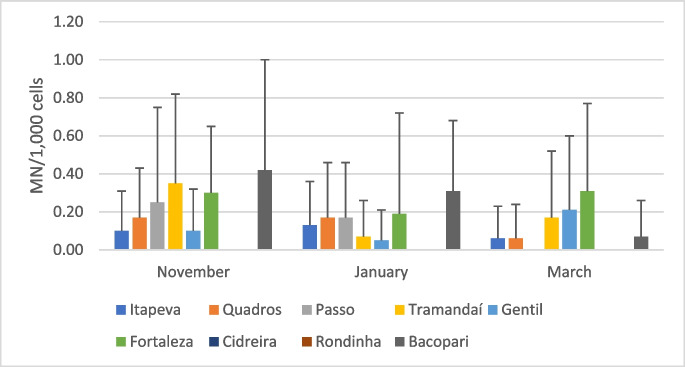
Micronucleus frequencies (mean ± standard deviation per 1,000 erythrocytes) in specimens of *G. iporangensis* captured in nine lagoons of the Tramandaí River Basin. Samples from Rondinha (November) and Cidreira (March) were not obtained. All other samples with no recorded values correspond to a frequency of zero

With respect to the evaluation of genotoxicity biomarkers, the alterations observed in the erythrocytes of *G. iporangensis* are shown in Fig. 2. A total of 168 individuals were examined (a detailed account of the number of specimens collected in each lagoon throughout the sampling periods is presented in Table 2). The mean frequencies of micronuclei (MN) per 1000 erythrocytes (‰) are presented in Fig. 3. MN frequencies ranged from 0.0 to 0.42‰, with no statistically significant differences detected between sampling periods at the same site or between sites within the same sampling period.

**Table 2:** Frequencies of nuclear abnormalities (per 1000 erythrocytes) in specimens of *G. iporangensis* captured in nine lagoons of the Tramandaí River Basin

Lagoon	November	January	March	p*
Itapeva	(10) 8.0 ± 3.4^A^	(8) 8.1 ± 4.9^A^	(9) 2.9 ± 4.1^B^	0.02
Quadros	(6) 9.8 ± 7.2	(3) 7.8 ± 3.8	(8) 6.2 ± 5.7	0.65
Passo	(4) 7.4 ± 2.4	(3) 6.7 ± 1.6	(2) 12.0 ± 7.8	0.55
Tramandaí	(10) 9.6 ± 5.6	(7) 2.9 ± 3.9	(9) 5.3 ± 5.8	0.07
Gentil	(5) 9.6 ± 4.4^A^	(10) 1.3 ± 2.0^B^	(7) 7.0 ± 7.4^A^	0.01
Fortaleza	(10) 6.6 ± 3.5^AB^	(8) 10.7 ± 6.6^A^	(8) 2.5 ± 3.5^B^	0.02
Cidreira	(5) 5.1 ± 4.3	(2) 3.0 ± 4.2	NC	0.69
Rondinha	NC	(5) 7.6 ± 7.5	(8) 10.5 ± 6.3	0.42
Bacopari	(6) 7.8 ± 4.8^AB^	(8) 12.3 ± 3.7^A^	(7) 3.3 ± 2.2^B^	0.003

The number of animals analyzed is shown in parentheses. NC: Not collected. *p value of the Kruskal–Wallis test; where *p* ≤ 0.05 the means with different uppercase letters indicate significant difference between sampling periods in the same sampling site

**Fig. 4:**
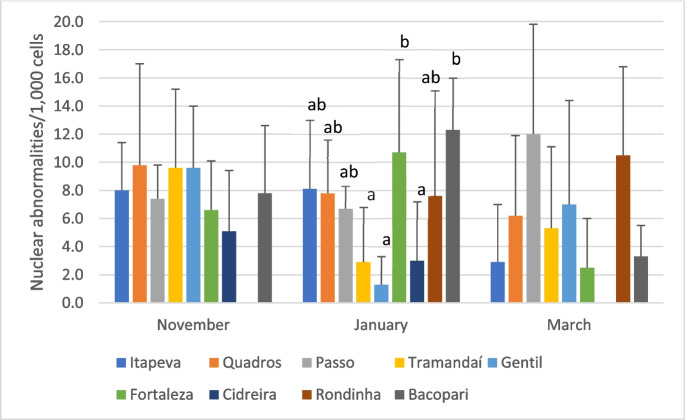
Analysis of the spatial variation in the frequencies of nuclear abnormalities (mean ± standard-deviation, per 1,000 erythrocytes) in specimens of *G.* *iporangensis* captured in nine lagoons of the Tramandaí River Basin. Lagoons labeled with different lowercase letters indicate significant differences across the sampling period (Kruskal-Wallis test; *p* ≤ 0.05)

In contrast, the frequencies of other nuclear abnormalities (NA) ranged from 1.3 to 12.3‰ and exhibit significant temporal variation in four lagoons (Table 2). NA frequencies were significantly higher in the peak vacation period (January) compared to post-vacation period (March) in the Itapeva (*p* = 0.02; η^2^ₕ = 0.22), Fortaleza (*p* = 0.02; η^2^ₕ = 0.25), and Bacopari (*p* = 0.003; η^2^ₕ = 0.61) lagoons. Conversely, the Gentil lagoon showed a significant decrease in NA frequency during January relative to the other two sampling periods (*p* = 0.01; η^2^ₕ = 0.35). Figure 4 presents the analysis of spatial variation in NA frequencies, showing significant differences among lagoons during the January sampling period. In this period, the Fortaleza and Bacopari lagoons exhibited significantly higher NA frequencies than the Tramandaí, Gentil, and Cidreira lagoons (*p* = 0.002; η^2^ₕ = 0.35).

The association between MN and NA frequencies and thermotolerant coliform levels across all sites and sampling periods was evaluated using Spearman correlation analysis. No significant correlation was found between TC levels and MN frequencies (r_S_ =− 0.22; p = 0,28), nor between TC concentrations and NA frequencies (r_S_ = − 0.11; *p* = 0,61).

## Discussion

The results of this study reveal a concerning pattern of microbiological contamination across the Tramandaí River Basin (TRB), as evidenced by elevated thermotolerant coliform levels in eight of the nine lagoons evaluated. High concentrations of thermotolerant coliforms constitute a clear indicator of fecal contamination (Dos Santos Mendonça et al., 2022). Coastal municipalities within the TRB experience pronounced seasonal population increases during warmer months, with some localities registering population growth exceeding 400% (Comitê de Gerenciamento da Bacia Hidrográfica do Rio Tramandaí, 2013). Such seasonal influxes, commonly observed in coastal regions worldwide, can overwhelm local wastewater treatment infrastructure and negatively affect water quality (Stanchev et al., 2015). Despite this context, the hypothesis that thermotolerant coliform concentrations would increase during the summer vacation period as a result of intensified domestic sewage inputs was not supported. A discernible influence of vacation periods on fecal contamination was observed only in the Passo and Fortaleza lagoons. In contrast, thermotolerant coliform concentrations frequently exceeded permissible limits in most water samples across the majority of lagoons, indicating widespread and persistent fecal pollution. The consistently elevated contamination levels observed in the Quadros, Tramandaí, Gentil, and Cidreira lagoons appeared to be independent of seasonal population fluctuations and may reflect continuous inputs of treated or untreated domestic sewage, runoff from agricultural or urban areas, and deficiencies in sanitary infrastructure (de Souza et al., 2025; Dos Santos Mendonça et al., 2022; Garbossa et al., 2017).

The widespread fecal contamination documented in the TRB lagoons represents a significant threat to aquatic biodiversity and poses substantial public health risks (Bashir et al., 2020). Recreational activities in contaminated waters are associated with increased incidence of waterborne diseases (Wade et al., 2022). Globally, swimming and bathing in polluted coastal waters are estimated to result in more than 120 million cases of gastrointestinal illness and approximately 50 million cases of severe respiratory disease annually (Gopinathan & Suthindhiran, 2025).

Among the lagoons studied, Itapeva Lagoon stood out, as all three samples met microbiological quality standards. This distinction may be attributed to lower anthropogenic pressure or more effective land-use management in its surrounding watershed. Previous studies demonstrate that integrated watershed management strategies, including riparian buffer preservation and efficient wastewater treatment, can substantially reduce bacterial contamination in aquatic ecosystems (Wang et al., 2017). Similar approaches may be required in the TRB to mitigate microbiological pollution more broadly.

All 27 water samples analyzed exhibited dissolved oxygen (DO) concentrations equal to or exceeding 6 mg/L, consistent with Brazilian standards for Class 1 waters, which represent the highest quality category intended for the protection of aquatic communities and primary contact recreation. Recent monitoring data corroborate these findings: according to FEPAM, analyses of water samples collected in 2022 across the TRB predominantly classified DO concentrations as Class 1 (FEPAM, 2023). These results suggest that, at the time of sampling, the lagoons maintained physicochemical conditions generally favorable to aquatic life (Bulbul and Mishra, 2022). The persistence of adequate DO levels despite high organic loads inferred from coliform concentrations may reflect the natural assimilative capacity of these systems, supported by self-purification processes and atmospheric reaeration (Pang & Guan, 2025; Xu et al., 2025). Nevertheless, DO alone failed to capture other relevant stressors, including fecal pollution and genotoxic contaminants, underscoring the limitations of single-parameter assessments and reinforcing the need for integrated assessments.

DNA damage assessments play a crucial role in identifying environmental threats and supporting the conservation of aquatic organisms (Andrés et al., 2025; Marques et al., 2025). The analysis of cytogenetic biomarkers in *G. iporangensis* provides additional insight into the sublethal biological effects of environmental stressors in the lagoons of the TRB, which had not previously been assessed. The micronucleus (MN) frequencies observed in the present study (0.0 to 0.42‰) did not exhibit statistically significant differences and fall within the lower range of values reported for *G. iporangensis* exposed to anthropogenic impacts in comparable freshwater environments. For example, Lehun et al. (2021) documented significant differences in MN frequencies (3.0 ± 0.6 to 7.7 ± 1.1‰) in specimens from lagoons classified as moderately to critically degraded, while de Campos Júnior et al. (2015, 2016) reported MN values ranging from 0.067 ± 0.018 to 0.627 ± 0.213‰, with statistically significant differences among sampling sites. These studies suggest that MN levels in *G. iporangensis* above approximately 0.5‰ are generally associated with higher contamination pressure, whereas values below this threshold, such as those found here, are typically interpreted as indicative of low to moderate genotoxic impact.

In contrast to the relatively low MN frequencies, nuclear abnormalities (NA) in our study ranged from 1.3 to 12.3‰ and displayed significant temporal and spatial variation, suggesting differential exposure to stressors across lagoons. Alterations in normal nuclear morphology, such as NA, are also considered indicators of genotoxic damage (Çavas and Könen, 2008; Omar et al., 2012; Furnus et al., 2014; Viana et al., 2017). According to Ghaffar et al. (2015), the analysis of NA represents one of the most effective diagnostic tools for assessing pollutant-induced genotoxicity in aquatic ecosystems. Comparable studies with the same species also report a higher sensitivity of NA to environmental gradients: Lehun et al. (2021) found NA frequencies between 1.1 ± 3.5 and 6.3 ± 6.4‰, with markedly elevated values at more degraded sites, while de Campos Júnior et al. (2015) observed NA levels ranging from 0.088 ± 0.057 to 3.965 ± 2.895‰. Similarly, de Jesus et al. (2016) reported significant differences between sites, with NA values from 3.00 ± 0.56 to 7.20 ± 1.16‰. Taken together, these comparisons suggest that although MN levels in the present study fall within ranges associated with relatively low genotoxic stress, the higher and more variable NA frequencies are consistent with environmental conditions characterized by moderate to substantial biological stress. These findings emphasize the importance of evaluating multiple cytogenetic biomarkers to contextualize the genotoxic status of aquatic ecosystems.

One of the hypotheses tested in the present study was that seasonal increases in human occupation and wastewater discharge during the summer vacation period would result in elevated MN and NA frequencies in resident fish populations.. However, the results from most lagoons did not support this expectation, largely due to the persistently high levels of fecal contamination observed throughout the sampling period. As a consequence, thermotolerant coliform concentrations were not correlated with the frequencies of genotoxic biomarkers. Evidence consistent with the seasonal hypothesis was observed in only three lagoons. In particular, NA frequencies were significantly lower in the post vacation period (March) in the Itapeva, Fortaleza, and Bacopari lagoons, suggesting that fish were exposed to reduced levels of genotoxic contaminants after the peak tourism period. Although this interpretation remains tentative, the decline in NA levels may indicate an improvement in water quality following the reduction in human occupation and associated pollutant inputs. These results align with previous studies showing that NAs are more sensitive than MNs to short term fluctuations in environmental stressors and pollutant exposure (Ayllon and Garcia Vazquez, 2000; Cavas and Ergene Gozukara, 2005).

The hypothesis that lagoons with persistently elevated fecal contamination would exhibit correspondingly higher cytogenetic damage, indicative of chronic exposure to genotoxic pollutants, was also not confirmed. In the Quadros, Tramandaí, Gentil, and Cidreira lagoons, thermotolerant coliform levels exceeded permissible limits during all three sampling periods; however, these lagoons did not exhibit higher MN or NA frequencies compared to the others. Although NA proved to be a sensitive biomarker for detecting genotoxic stress in the lagoons of the TRB, the significant spatial and temporal variation observed in this response could not be explained by differences in coliform concentrations or by persistently elevated fecal contamination. Given the diversity of pollutant sources affecting these lagoons (Fonseca et al., 2025; Pagani et al., 2022; Tesser et al., 2021), future environmental assessments will require a finer contextualization of cytogenetic responses within the specific ecological settings of each lagoon. This includes considering the potential influence of episodic pollution events, differences in water retention time, and lagoon-specific land-use patterns (Asllani et al., 2024; Varea & Mani, 2024).

Some limitations of this study should be acknowledged. The sex and age of sampled fish were not determined, precluding evaluation of potential biological influences on cytogenetic responses. Additionally, the absence of chemical contaminant analyses limited the ability to directly link observed genotoxic effects to specific pollutants. Logistical constraints resulted in missing samples during two sampling events, and some groups exhibited small sample sizes (*n* = 2–3), reducing statistical power. Nevertheless, in all cases where significant differences in NA were detected using the Kruskal–Wallis test, effect sizes calculated as ordinal eta-squared (η^2^ₕ) indicated large effects, reflecting substantially different data distributions among groups (Fiel Peres, 2026). Taken together, these factors should be carefully considered when interpreting the results and drawing conclusions regarding environmental impacts on the studied fish populations and lagoon systems.

## Conclusions

Regarding dissolved oxygen (DO), all lagoons met the highest-quality category established for the protection of aquatic communities, indicating favorable physicochemical conditions at the time of sampling. In contrast, fecal contamination, as evidenced by thermotolerant coliform concentrations, revealed a more concerning and heterogeneous scenario. Eight of the nine lagoons exceeded the permissible limits for primary contact recreation in at least one sampling event, and four lagoons remained unsuitable for such use throughout all sampling periods.

When considered jointly, the water quality parameters and cytogenetic biomarkers assessed in the nine lagoons of the TRB did not exhibit a consistent seasonal pattern. The influence of increased human occupation during vacation periods was detectable only in a subset of lagoons, being reflected in elevated fecal contamination in two lagoons and higher genotoxic responses in four lagoons. Cytogenetic damage, particularly as indicated by nuclear abnormalities, displayed marked spatial and temporal variability, suggesting that genotoxic stress in these systems is influenced by lagoon-specific conditions rather than by seasonal population increases alone.

Although sewage effluents are recognized sources of genotoxic substances, no clear relationship was observed between thermotolerant coliform concentrations and cytogenetic damage in resident fish populations. This lack of association indicates that fecal contamination alone is not a reliable proxy for genotoxic stress in these lagoon systems and highlights the likelihood that multiple, diffuse, and temporally variable contamination sources contribute to the observed biological responses.

Taken together, these findings emphasize the complexity of environmental pressures acting on coastal lagoon ecosystems within the TRB and underscore the importance of integrated monitoring approaches that combine microbiological, physicochemical, and biological indicators. While the results point to potential ecological and public health concerns associated with the co-occurrence of fecal contamination and genotoxic stress, definitive causal links could not be established within the scope of this study. Consequently, future investigations incorporating chemical contaminant analyses and lagoon-specific contextual factors are necessary to better elucidate the drivers of genotoxic effects in these environments.

## Data Availability

No datasets were generated or analysed during the current study.

## Declarations

All authors have read, understood, and have complied as applicable with the statement on “Ethical responsibilities of Authors” as found in the Instructions for Authors.
